# ‘Nothing to lose or a world to win’: Reconsidering efficacy, legitimacy, political trust and repression in confrontational collective action

**DOI:** 10.1111/bjso.12891

**Published:** 2025-04-28

**Authors:** Mete Sefa Uysal, John Drury, Yasemin Gülsüm Acar

**Affiliations:** ^1^ Department of Psychology University of Exeter Exeter UK; ^2^ School of Psychology University of Sussex Brighton UK; ^3^ School of Psychology and Neuroscience University of St. Andrews St. Andrews UK

**Keywords:** confrontational collective action, efficacy, legitimacy, political trust, protests, repression

## Abstract

Confrontational collective actions are neither uncontrolled outbursts of initially pacifist resistance nor mere reactions to helplessness and lack of viable political options. Instead, they serve strategically determined purposes within the group, making them perceived as both effective and legitimate. Regardless of whether it is more or less confrontational, examining the role of efficacy and legitimacy of actions that are committed to achieving group goals is crucial for understanding the appeal of collective action strategies. We examined the role of political trust and protest repression in predicting the legitimacy of protest violence and the perceived efficacy of confrontational and non‐confrontational collective actions and, in turn, their role in confrontational collective action. Across three correlational studies conducted in Germany, Turkey and the United Kingdom (*N* = 3833), the legitimacy of protest violence and the efficacy of confrontational tactics were core determinants of confrontational collective actions. While low political trust did not directly predict confrontational action, it predicted heightened protest repression and the legitimacy of protest violence. Our findings challenge the *nothing‐to‐lose hypothesis* by demonstrating that confrontational actions are not driven by the low efficacy of non‐confrontational strategies or low political trust, and people may perceive both confrontational and non‐confrontational actions as similarly effective.

## INTRODUCTION

The majority of the current models and studies on collective action are based on non‐confrontational actions, such as street protests and demonstrations that do not involve outgroup action, disruptions, counter‐protests or the potential political or physical risks following engagement in the actions (Agostini & van Zomeren, 2021; Greijdanus et al., 2020; Prentice & Paluck, 2020; Thomas et al., 2012; van Zomeren et al., 2008). However, confrontational collective actions, variously defined as non‐normative, radical, aggressive, or violent collective actions as they may utilize civil disobedience, disruptive activism, protest violence and conflict escalation, are increasingly coming into the focus of social psychological studies (see Uysal et al., 2025; Uysal, Saavedra, & Drury, 2024). In this study, we use the term ‘confrontational collective action’ to address overlapping concepts in the literature (e.g. radical, aggressive, high‐risk, violent, non‐normative) and to reduce conceptual ambiguity (see Uysal, Saavedra, & Drury, 2024).

*Confrontational collective action* refers to forms of actions that involve direct challenges, use of force, or physical opposition, typically characterized by one or more of the following: (i) physical conflict between protesters and the police, (ii) the threat or likelihood of police intervention and/or (iii) participation that entails legal and political sanctions or the expectation thereof. With this conceptualization, we avoid using social approval by the majority to name and explain a form of action strategy, which is inherent in the current dichotomies of the literature, such as normative versus non‐normative.

This conceptualization is not intended to introduce a new dichotomy to existing ones (e.g. normative vs. non‐normative or moderate vs. radical) but rather to frame confrontational and non‐confrontational actions as possible points along a continuum of collective action participation. Groups in protests often do not see confrontational and non‐confrontational action as mutually exclusive strategies as most studies in social psychology do; rather, they approach them as part of a diverse and dynamic range of political strategies, employing both strategies simultaneously or at different times (Penić et al., 2024; Uysal et al., 2025; Uysal, Saavedra, & Drury, 2024; Zúñiga et al., 2023). Therefore, we acknowledge the potential interdependence and fluidity between these strategies within the broader repertoire of collective action. In the political repertoire where groups simultaneously or at different times employ both types of strategies, the perceived legitimacy and efficacy of these strategies could emerge as core determinants of individuals' willingness to engage in confrontational action or shift their tactics towards more confrontational ones in response to situational and intergroup dynamics. Diminished political trust and perceived political repression are some of the most relevant variables related to situational and intergroup dynamics that can facilitate confrontational collective action or such tactical shifts (Saavedra & Drury, 2019; Tausch et al., 2011). Therefore, across three cross‐sectional survey studies conducted in Germany, Turkey and the United Kingdom, we examined the role of the efficacy of confrontational and non‐confrontational tactics, the legitimacy of protest violence, political trust and perceived protest repression in the confrontational collective action.

### Reconsidering efficacy

Confrontational collective actions can entail significant individual and collective risks (Ayanian et al., 2021; Jiménez‐Moya et al., 2015; Uysal et al., 2022, 2025), and engagement in these actions may require a robust psychological commitment justified by moral or normative reasoning in line with the ingroup norms and strategic collective goals. Therefore, rather than being reflections of ‘mob mentality’ (as characterized by early crowd psychologists) and uncontrolled outbursts of initially non‐confrontational actions or consequences of helplessness and lack of options (as suggested by some contemporary collective action researchers), these actions are increasingly understood as having particular meanings, methods and purposes that are determined and shared by the ingroup (Ayanian et al., 2025; Drury et al., 2012; Drury & Reicher, 1999; Penić et al., 2024; Reicher, 1996; Uysal et al., 2025; Uysal, Saavedra, & Drury, 2024; Zúñiga et al., 2023). Hence, to engage in confrontational collective action, people must see it as legitimate and as significantly contributing to the group's goals.

Psychological theory and research have emphasized the pivotal role of human agency and the belief in the capacity of individuals or collectives to achieve specific goals in motivating collective action (Mummendey et al., 1999; van Zomeren et al., 2008). While the mobilizing impact of collective efficacy, which has been defined as an individual's belief in their ability to address group‐related issues through collective efforts (Mummendey et al., 1999), has been extensively studied in social psychology concerning non‐confrontational collective action, its relevance in confrontational collective action remains relatively underexplored and there are inconsistencies in existing findings (for a review, see Uysal, Saavedra, & Drury, 2024).

A recent review revealed that some of these inconsistencies arise from a lack of clarity in the conceptualization and operationalizations of efficacy in the collective action literature: it argued that which agents, actions and aims are involved is crucial for the conceptualization and measurement of efficacy (Hamann et al., 2024). The authors showed that there is an overabundance of efficacy labels that claim to measure the same (or overlapping) concept(s) and its effect on collective action. The inconsistencies go beyond the labelling of the concept and signal a broader conceptualization problem. The review highlighted that most research in social psychology overlooks the fact that efficacy beliefs emerge from the interplay of beliefs about an individual or collective agent, their behaviour and a specific outcome (Hamann et al., 2024). The three core features of efficacy beliefs can be linked in four possible ways (p. 7): (a) agent and action (e.g. *we can organize a sit‐in*), (b) action and aim (e.g. *peaceful protests can change the policy*), (c) agent and aim (e.g. *we can stop deforestation*) and (d) agent, action and aim (e.g. *we can save animals by occupying slaughterhouse*).

This review demonstrated that while most studies conceptualie and measure efficacy through the agent‐aim link (e.g. *we, as members of Greenpeace, can promote environmental justice*), relatively fewer studies can effectively connect a particular intentional action to that agent‐aim link of efficacy (i.e. agent‐aim‐action link; e.g. *we, as members of Greenpeace, can promote environmental justice by distributing a petition*). Thus, by leveraging insights from the recent reviews (Hamann et al., 2024; Uysal, Saavedra, & Drury, 2024), we posit that the perceived efficacy of specific tactics or actions, dedicated to specific group goals, and enacted by a clearly defined ingroup, predicts confrontational collective actions.

### Relationship between efficacy and legitimacy

Efficacy and legitimacy are considered analytically distinct concepts explaining collective action in both Social Identity Theory (Tajfel & Turner, 1979) and the Elaborated Social Identity Model (Drury & Reicher, 2000, 2009; Reicher, 1996; Stott & Reicher, 1998). However, an action's perceived effectiveness in achieving desired social change can be crucial for attaining political credibility and acceptance. This principle underlies counter‐mobilization efforts that seek to prevent outgroups from organizing and demonstrating publicly while also creating more platforms for ingroups (Hoerst & Drury, 2023). When an organization is perceived as capable of realizing its beliefs, it is seen as a more legitimate political force (Reicher & Haslam, 2006). Research on bystander support for social movements indicates that the perceived efficacy of an action influences its legitimacy. Jimenez‐Moya et al. (2019) argue that the belief in the achievability of social change leads people to attribute efficacy to social movements, viewing their actions as useful and functional, which in turn enhances their legitimacy.

In line with these arguments, we suggest that legitimacy is not a fixed attribute: the appropriateness of a tactic is evaluated based on its potential to achieve its specific objectives. For example, a protest movement aimed at drawing urgent attention to climate change might view disruptive actions, such as blocking traffic, as legitimate because these actions effectively raise awareness and put pressure on policymakers. In contrast, in a different context where the action group aims to dialogue with policymakers and the broader public, the same disruptive tactics might be viewed as less legitimate, since they might not be effective in achieving such goals. When people believe that a particular action is effective in achieving goals, they are more likely to view that action as legitimate. Hence, we argue that high tactic efficacy will be associated with high legitimacy. In turn, this combined perception of high efficacy and legitimacy predicts a stronger intention to engage in confrontational collective action.

### The ‘nothing‐to‐lose’ hypothesis

Becker, Tausch, and colleagues (Becker & Tausch, 2015; Tausch et al., 2011) argued that low collective efficacy motivates confrontational collective action. Following this argument, the ‘nothing‐to‐lose’ approach (Jiménez‐Moya et al., 2015; Saab et al., 2016; Tausch et al., 2011) posits that individuals engage in confrontational actions when they perceive no alternative means for political change, or in other words, have low group efficacy. Yet, there is little consistent empirical evidence to support this claim on the one hand (see Uysal, Saavedra, & Drury, 2024); and there are only a few studies that test this hypothesis by conceptualizing efficacy through tactic efficacy (see agent‐aim‐action link, Hamann et al., 2024), on the other hand.

In one of the few studies focusing on the efficacy of specific actions (e.g. efficacy of confrontational vs. non‐confrontational tactics), rather than the collective efficacy of agents (e.g. self‐efficacy, group efficacy), Saab et al. (2016, Study 2) suggested that Palestinians' perceived effectiveness of confrontational actions in pressuring Israel to make compromises predicted their intention to engage in confrontational collective action, irrespective of the perceived efficacy of non‐confrontational actions. In other words, individuals who perceive confrontational collective actions as effective in achieving certain collective goals may adopt such actions, even if they believe non‐confrontational resistance could also be fruitful. There is high efficacy associated with confrontational collective action, alongside the high levels of perceived efficacy of non‐confrontational collective action. Therefore, not only confrontational and non‐confrontational collective actions themselves, but the high efficacy of these tactics can co‐exist simultaneously. Thus, we tested the role of the efficacy of both tactics in confrontational collective action.

### Political trust

Political trust reflects an individual's evaluation of whether the political system operates within their normative expectations, moral judgements and situational preferences (Devine et al., 2020; Levi & Stoker, 2000). It encompasses confidence in the fairness, integrity and responsiveness of political institutions and actors, shaping the extent to which individuals perceive these entities as legitimate and capable of addressing societal concerns (Hetherington, 2005; Hetherington & Husser, 2012). High levels of political trust play a critical role in maintaining the status quo, as they are positively associated with supporting incumbent political actors and their political actions (Kogler et al., 2013; Li, 2011). Conversely, political distrust arises when individuals perceive that political institutions fail to meet their normative expectations, fostering feelings of alienation and dissatisfaction with the existing political system.

Low political trust has been linked to engagement in confrontational or radical forms of collective action, particularly in high‐risk or repressive political contexts. For instance, in Hong Kong, low political trust predicted both collective action engagement and support for defensive violent collective action (Gulliver et al., 2023). Similarly, Li and Finkenauer (2021) demonstrated that low political trust in Hong Kong was associated with an increased acceptance of aggression towards the police. In Turkey, Acar and Uluğ (2022) found a negative relationship between political trust and protest participation, particularly in a high‐risk political environment. These findings align with the broader theoretical claim (see nothing‐to‐lose hypothesis) that political distrust motivates individuals to seek change outside of conventional channels, as they perceive these channels to be ineffective or compromised (Becker & Tausch, 2015; Scheepers et al., 2006). While discussing the role of low efficacy in driving radical collective actions, Tausch et al. (2011) argued that low efficacy also indicates low political trust, which could be seen as indicative of a perception that the political system is closed or unresponsive, leading individuals to reject moderate forms of engagement (e.g. voting, authorized or low‐risk protests) in favour of more confrontational tactics. Hence, this perspective indicates that confrontational forms of collective action should be driven by the low efficacy of non‐confrontational tactics and low political trust. We tested these assertions of the *nothing‐to‐lose hypothesis* by examining the direct and indirect (via efficacy and legitimacy) role of political trust in confrontational collective action.

### Protest repression

While repression of collective action has traditionally been studied as a macro‐level objective factor in social sciences, with mixed findings regarding its effects—deterring participation (Boykoff, 2006; Wood, 2007), mobilizing it (Adam‐Troian et al., 2020; Ayanian & Tausch, 2016; McAdam, 1990) or prompting tactical shifts in resistance strategies (Francisco, 1995; Lichbach, 1987)—more recent work has argued for a focus on individual perceptions of repression (Honari, 2018; Saavedra & Drury, 2019; for multi‐level review, see Ayanian et al., 2025). Honari (2018) emphasized that repression's effects on political participation vary significantly depending on how individuals perceive and interpret it. Even when embedded within similar macro‐structural contexts, individuals may evaluate the same objective conditions of repression differently, leading to diverse perceptions and responses. This discrepancy highlights the importance of studying perceived repression, which might not align directly with objective measures of state repression, as individuals' subjective interpretations shape their reactions to it.

In social psychology, research examining collective action in repressive contexts remains limited. Existing studies have primarily explored the perceived likelihood of risks related to repression, such as the subjective risk of arrest or police violence. For instance, Ayanian et al. (2021) found that the perceived likelihood of such risks could fuel collective action through anger. Similarly, recent findings suggest that the perceived likelihood of repression in the climate movement context predicts confrontational but not non‐confrontational climate collective action (Uysal et al., 2025). However, other studies also suggest that the perceived likelihood of such risks can dampen the impact of other motivators, such as moral obligation, for engaging in high‐risk collective actions (Uysal et al., 2022). Other studies in social psychology have either focused on the moderating role of repression as an objective macro‐level variable as in social science (Uysal, Vestergren, et al., 2024) or explored the relationship between perceived repression and conventional political participation (Abrams et al., 2020; Grant et al., 2017). However, examining the role of perceived repression in collective action requires a focus on domain‐specific repression: perceived protest repression (see Saavedra & Drury, 2019).

The tactical shift approach to repression (Francisco, 1995; Lichbach, 1987; Moghadam & Gheytanchi, 2011) suggests that when repression—or the perception of it—is high, individuals may adopt more confrontational or alternative tactics to challenge the system (for non‐organized resistance tactics under oppression, see Marazzi & Vollhardt, 2025). Perceived protest repression is thus expected to positively predict the legitimacy of protest violence and the efficacy of confrontational collective action. Moreover, perceived protest repression is conceptually linked to political trust. A higher perception of repression often corresponds with diminished political trust, as repression signals a breakdown in the perceived legitimacy and fairness of political institutions.

### Current research and hypotheses

Overall, we examined the role of the efficacy of different tactics (confrontational and non‐confrontational) in confrontational collective action. To further examine the *nothing‐to‐lose hypothesis*, we specifically tested the role of political trust and the efficacy of non‐confrontational tactics in confrontational collective action, arguing that political trust and the efficacy of non‐confrontational action should negatively correlate with confrontational collective action if individuals engage in confrontational tactics due to a perception that the political system is closed and non‐confrontational action is not a viable option. Therefore, we investigated whether confrontational collective action is predicted by low political trust, high protest repression and the weak efficacy of non‐confrontational tactics (e.g. signing petitions, authorized demonstrations), aligning with the *nothing‐to‐lose hypothesis*. Contradicting these assertions, we hypothesised that the efficacy of confrontational tactics and the legitimacy of protest violence are the core determinants of confrontational collective action and tested whether confrontational collective action is predicted by stronger efficacy of confrontational tactics and the legitimacy of protest violence.

**FIGURE 1:**
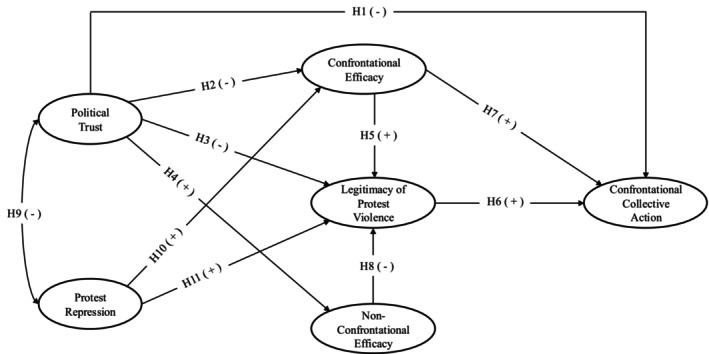
Conceptual Model and Summary of Hypotheses.

To test these hypotheses, we conducted three correlational studies in three socio‐political contexts in Turkey, Germany and the United Kingdom. Germany was included as a case for broader generalizability, utilizing a large representative community sample to examine the predictors of confrontational collective action in a relatively stable socio‐political environment. Turkey represents a context of long‐standing ethnopolitical conflict and state oppression, exemplified by the case of the Kurdish resistance. The United Kingdom provides a contrasting case, focusing on the Scottish independence movement, which operates within a framework of democratic processes but under perceived economic and political inequality. Building on the above theorization, we test the following hypotheses (see Figure 1):

Hypothesis 1–4 *Political trust will predict confrontational collective action, the efficacy of confrontational action and the legitimacy of protest violence negatively, while it will predict the efficacy of non‐confrontational collective action positively*.

Hypothesis 5 *The efficacy of confrontational collective action will predict greater legitimacy of protest violence*.

Hypothesis 6 *Legitimacy of protest violence will positively predict confrontational collective action*.

Hypothesis 7 *The efficacy of confrontational collective action will positively predict confrontational collective action*.

Hypothesis 8 *The efficacy of non‐confrontational collective action will negatively predict the legitimacy of protest violence*.

Hypothesis 9 *Political trust will be negatively correlated with protest repression (Study 3)*.

Hypothesis 10 *Perceived protest repression will positively predict the efficacy of confrontational collective action (Study 3)*.

Hypothesis 11 *Perceived protest repression will positively predict the legitimacy of protest violence (Study 3)*.

## STUDY 1

### Method

#### Participants

In the first study, we used secondary data from the German General Social Survey (GGSS). The datasets and analysis codes are openly accessible at https://osf.io/zysf7/?view_only=59107f659aa2422eb3c04a337f1ff6e8.

GGSS is a nationally representative panel survey project. For the present study, we utilized the data collected in 2018, which was published as ALLBUS 2018 (GESIS, 2019). The original dataset initially included 3477 participants. After excluding 62 participants who did not respond to the items related to our variables, the final sample comprised 3415 participants, consisting of 1673 females and 1742 males. The average age of the participants was 51.72 (SD = 17.58).

#### Measures

##### Political Trust

Political trust was assessed using three items. Participants were prompted to evaluate their level of trust in specific institutions or organizations, using a scale ranging from 1 (*no trust at all*) to 7 (*a great deal of trust*). The evaluated entities included the ‘*German government*,’ ‘*German parliament*,’ and ‘*political parties*.’ (*α* = .84).

##### Legitimacy of using political violence

It was measured with a single item that reads as ‘*Violence can be morally justifiable in order to achieve certain political goals*’ (1 = *do not agree at all*; 4 = *completely agree*).

##### Tactic efficacy

To measure the efficacy of confrontational and non‐confrontational collective action strategies, participants were presented with the prompt, ‘*If you wanted to have political influence or to make your point of view felt on an issue, which of the possibilities listed below would you use?*’ Within a set of political actions (such as voting in elections, engaging in voluntary work for a political party, launching an online petition, boycotting goods for political reasons, etc.), participants selected items they perceived as efficient for instigating political change. The chosen items were coded as ‘1’ while non‐selected items were coded as ‘0’. For assessing the efficacy of non‐confrontational collective action, we utilized the item ‘*I would take part in an authorized demonstration*.’ Conversely, to assess the efficacy of confrontational collective action, the item ‘*I would take part in an unauthorized demonstration*’ was employed.

##### Confrontational collective action

Participants were asked, ‘*Which of the following political actions have you done before, what have you already taken part in?*’ Participants chose items they have taken part in among a group of political actions (e.g. vote at elections, voluntary work for a political party, launch your own online petition, boycott goods for political reasons). The selected items were coded as ‘1’ and non‐selected items were coded as ‘0’. *Participated in unauthorized demonstrations* before is taken as confrontational collective action.

We also measured gender, age, region (i.e. the west vs. east part of Germany), social class, economic situation, and political orientation as control variables (see Data S1).

### Results and discussion

**FIGURE 2:**
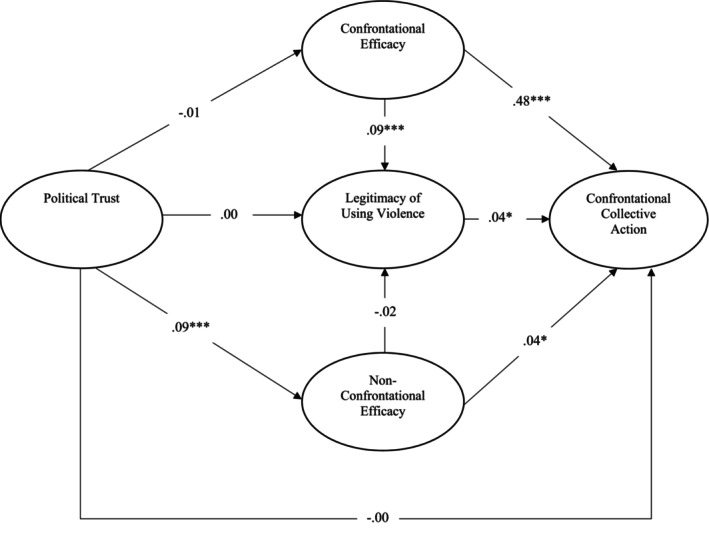
Summary of structural equation model, Study 1. *p < .05, ***p < .001.

Correlations between variables are depicted in Table 1. We conducted structural equation modelling (SEM) to test our hypotheses, utilizing the *lavaan* package in *R* (Rosseel, 2012). In the overall assessment, the fit indices indicated that our model demonstrated a good fit with the data: χ^2^ = 202.011; df = 9; *p* < .001; CFI = .964; TLI = .915; RMSEA = .080, CI [.070, .090]; and SRMR = .05. As illustrated in Figure 2, greater efficacy of non‐confrontational collective action was predicted by stronger political trust (*b* = .09, SE = .01, *p* < .001). However, political trust did not predict the efficacy of confrontational collective action, the legitimacy of using political violence, and participation in confrontational collective action. The efficacy of confrontational collective action positively predicted greater legitimacy of using political violence (*b* = .09, SE = .04, *p* < .001). Lastly, confrontational collective action was predicted by a higher legitimacy of using political violence (*b* = .04, SE = .01, *p* = .013) and stronger efficacy of confrontational (*b* = .49, SE = .01, *p* < .001) and non‐confrontational collective action (*b* = .04, SE = .01, *p* = .011).

**TABLE 1:** Means, standard deviations and correlations of all measures, Study 1.

	M	SD	1	2	3	4	5
1. Political trust	3.84	1.19	–	.00	.08***	−.01	−.01
2. Legitimacy of using political violence	1.41	.69		–	−.00	.09***	.08***
3. Efficacy of non‐confrontational action	.47	.50			–	.23***	.15***
4. Efficacy of confrontational action	.09	.28				–	.50***
5. Confrontational collective action	.05	.21					–

*Note*: ***p < .001; response scales: political trust (1–7), legitimacy of political violence (1–4), efficacy of strategies (0–1), confrontational collective action (0–1).

As a robustness check, we conducted an alternative model where we switched the roles of efficacy and legitimacy. In other words, in the alternative model, efficacy beliefs are predicted by legitimacy, as opposed to the original model where efficacy beliefs predicted legitimacy. Results showed that the original model had a lower *AIC* and *χ* ^*2*^ and higher *CFI*, *GFI* and *AGFI*, suggesting the proposed model showed a better fit than the alternative model (see Data S1). Therefore, tactic legitimacy is more likely to be predicted by tactic efficacy, compared to the other way around.

Finally, we conducted an explanatory mediation analysis to examine whether the efficacy of confrontational collective action indirectly predicts confrontational collective action through the legitimacy of using political violence. Utilizing a bootstrapping method with 10,000 repetitions, our findings revealed that the efficacy of confrontational action directly and positively predicted confrontational collective action. However, no significant indirect effect through the legitimacy of political violence was observed.

Thus, we confirmed our hypotheses that confrontational collective action is predicted by both the legitimacy of using political violence and the efficacy of confrontational and non‐confrontational tactics. Our findings highlight that the efficacy of confrontational tactics emerged as the most potent predictor of engaging in confrontational collective action. We also observed that the legitimacy of using violence is positively predicted by the efficacy of confrontational collective action. Furthermore, Study 1 reveals that political trust is only associated with the efficacy of non‐confrontational collective action, while no significant relationship was identified between political trust and confrontational collective action, the efficacy of confrontational tactics, or legitimacy, contradicting hypotheses informed by the *nothing‐to‐lose* approach. However, this might be attributed to the relatively non‐conflictual nature of Study 1 and the political landscape in Germany at the time of data collection. It is plausible to argue that in more conflicted contexts, political trust might exhibit a negative relationship with confrontational strategies within political opposition. Finally, participants were not identified with specific political or social groups, and ingroup identification and norms were not directly relevant to confrontational collective action in this context. It is important to replicate and extend these findings in contexts where ingroup identification and group norms inform the tactical choice of activists within sustained mobilization.

## STUDY 2

We replicated and extended these findings using primary data and more context‐specific measurements in a more conflictual and repressive political context: the Kurdish movement in Turkey. By doing so, in contrast to Study 1 which concentrated on a single form of confrontational collective action (i.e. unauthorized demonstration), Study 2 measured confrontational collective action using a multi‐item survey encompassing various forms of confrontational actions, such as occupying, road‐closing, damaging properties, throwing rocks, etc. Similarly, in Study 1, legitimacy was measured with a single item, broadly focusing on political violence. In Study 2, we measured the legitimacy of using violence within the context of collective action specifically, employing a multi‐item scale that examines diverse political goals for which protest violence may be employed (e.g. protesting repression, challenging police brutality, gaining national independence). We also measured the efficacy of confrontational collective action with a multi‐item measure, extending beyond being efficient to ‘have a political influence.’ Last, while the first study focused on past protest participation, Study 2 shifted the focus to participants' willingness to engage in future collective actions as an outcome variable.

State oppression against Kurds and Kurdish resistance in Turkey is a long‐standing issue characterized by asymmetrical violence, non‐recognition and colonization within the Turkish Republic (Acar et al., 2025; Şen et al., 2023; Uysal et al., 2024a, 2024b). Despite Kurds comprising around 20% of the population, they have never been formally recognized as a minority by the Turkish state (Koc et al., 2008; Mutlu, 1996). The conflict has manifested in various Kurdish uprisings throughout Turkish history, sparked by attempts at cultural assimilation and colonization through Turkification policies (Cagaptay, 2004). The Kurdish movement, originating as a left‐wing movement in the 1960s against state oppression, later evolved into an ethnic independence movement in the late 1970s. It eventually transformed into a minority rights movement advocating for fundamental rights such as freedom for Kurdish politicians, Kurdish language education, an end to state operations in Kurdistan, and demands for direct democracy and pluralism (Grigoriadis, 2016; Güneş, 2019). In this context, Study 2 aimed to explore the extent to which political trust, legitimacy of using *protest* violence and tactic efficacies predict the Kurds' intention to participate in confrontational collective action in Turkey.

### Method

We collected data from self‐identified Kurdish participants in Turkey. To determine the minimum number of observations required for examining the research hypotheses, we conducted a power analysis using G*Power 3.1, considering the smallest correlation coefficient between predictors and confrontational collective action in the first study (*r*_legitimacy_ = .08). With an *α* value of .05, a power of .80 and a one‐way null hypothesis, the analysis indicated that a minimum of 109 observations would be necessary to perform linear regression analysis with four predictors.

Data collection took place between March and June 2022. We distributed the survey link through the first author's social media accounts and collaborated with NGOs working for Kurdish and human rights in Turkey. A total of 114 participants completed our survey. Among them, 61 identified as male, 37 as female, 7 as non‐binary and nine as other or preferred not to answer. Participants' ages ranged from 20 to 72 (*M* = 34.52, SD = 10.48). In terms of residence, 29 participants lived in Kurdish‐majority cities in Turkey, 67 in the three largest cities of western Turkey (39 in Istanbul, 15 in Izmir and 13 in Ankara), 15 in other western cities and three participants did not provide location information.

### Measures

We used 5‐point response scales (1 = *strongly disagree*; 5 = *strongly agree*) unless otherwise specified.

#### Political trust

Trust was assessed with four items. Items read as ‘*I trust politicians/political parties/parliament/government in Turkey*’ (*α* = .81).

#### Legitimacy of protest violence

Participants' attitudes towards the use of protest violence to achieve certain political goals were measured with five adapted items (Jackson et al., 2013). Participants evaluated how right or wrong they believe it is to use violence for a set of political goals (1 = *always wrong*, 5 = *always right*). Items were *using violence to protest ‘police brutality*,’ ‘*state repression*,’ ‘*government's unjust policies*’ and ‘*crimes of the government*’ (*α* = .96).

#### Efficacy of confrontational tactics

The perceived efficacy of confrontational collective actions was measured with four items adapted from the group efficacy scale of van Zomeren et al. (2010) and the participative efficacy scale of van Zomeren et al. (2013). Items were ‘*Kurds can challenge oppression through violent protests*,’ ‘*Kurds can establish a free life together through violent protests*,’ ‘*I can make an important contribution to the Kurdish movement by participating in violent protests*’ and ‘*I can provide significant support to Kurdish resistance by participating violent protests*’ (*α* = .92).

#### Efficacy of non‐confrontational tactics

Similarly, we measured perceived efficacy of non‐confrontational tactics with four adapted items (van Zomeren et al., 2010, 2013). Items were ‘*Kurds can challenge the oppression through non‐violent pacifist protests*,’ ‘*Kurds can establish a free life together through non‐violent pacifist protests*,’ ‘*I can make an important contribution to the Kurdish movement by participating non‐violent pacifist protests*’ and ‘*I can provide significant support to Kurdish resistance by participating non‐violent pacifist protests*’ (*α* = .87).

#### Confrontational collective action

We measured participants' willingness to participate in confrontational collective action with five items. Participants were asked to indicate their intention to participate in different collective actions in future to protest the Turkish government's policies and repression against Kurds: *occupying*, *road‐closing*, *participating in unauthorized demonstrations*, *throwing rocks at police* and *damaging properties* (*α* = .93).

### Results and discussion

The descriptive statistics and zero‐order correlations among our variables are detailed in Table 2.

**TABLE 2:** Means, standard deviations and correlations of all measures, Study 2.

	M (SD)	1	2	3	4	5
1. Political trust	1.55 (.61)	–	−.31**	−.01	−.34***	−.30**
2. Legitimacy of protest violence	2.64 (1.40)		–	−.42***	.74***	.77***
3. Efficacy of non‐confrontational tactics	3.31 (1.15)			–	−.26**	−.28**
4. Efficacy of confrontational tactics	2.49 (1.20)				–	73***
5. Confrontational collective action intention	2.49 (1.25)					–

*Note*: ***p < .001, **p < .01.

Due to the strong correlations between variables, we performed multicollinearity diagnostics. Results indicated that multicollinearity was not a concern (Tolerance [.38, .86], VIF [1.26, 2.61]). Subsequently, we conducted a linear regression analysis to assess the extent to which political trust, legitimacy of protest violence and efficacy of confrontational and non‐confrontational tactics predict the willingness to participate in confrontational collective action among Kurds in Turkey while controlling for age and education. Similar to the findings in the first study, political trust (*b* = −.05, *p* = .702) did not predict confrontational collective action. Stronger legitimacy of protest violence (*b* = .45, *p* < .001) and greater efficacy of confrontational collective action (*b* = .37, *p* < .001) emerged as significant positive predictors. No significant relationship was found between the efficacy of non‐confrontational tactics and the willingness to participate in confrontational collective action (see Table 3).

**TABLE 3:** Model summary of regression analysis in Study 2.

Confrontational collective action intention					
	*b*	SE	*β*	*t*	*p*
Political trust	−.05	.13	−.02	−.38	.702
Legitimacy of protest violence	.45	.08	.51	5.56	<.001
Efficacy of non‐confrontational tactics	.03	.07	.03	.47	.641
Efficacy of confrontational tactics	.37	.09	.36	4.12	<.001
*F*	50.721				
*R* ^*2*^	.65				

Similar to Study 1, Study 2 did not reveal a significant relationship between political trust and confrontational collective action. However, we replicated the findings on the role of the legitimacy of protest violence and the efficacy of confrontational actions. The legitimacy of protest violence and the efficacy of confrontational collective action emerged as significant predictors of confrontational collective action intention. It is crucial to note that the study faced limitations in terms of statistical power, and the observed findings should be interpreted with caution. Replication of this study with a more substantial sample size is essential to enhance the robustness and generalizability of the results. Despite this limitation, the current study emphasized the significance of the efficacy and legitimacy of confrontational tactics as proximal predictors of confrontational collective action in a repressive and non‐democratic conflict context. In the next study, we aimed to replicate and extend these findings in a conflict context within a democratic regime, ensuring an adequate sample size for more reliable results.

## STUDY 3

In Study 3, our focus shifts to the Scottish independence movement in the United Kingdom. Previous research by Abrams and Hogg (1987) highlighted that Scottish adolescents tended to support Scottish independence when they perceived that the UK government was extracting resources (e.g. North Sea oil) from Scotland, coupled with the belief that Scotland was economically repressed by England. More recent studies, such as Abrams et al. (2020), found that, prior to the 2014 Scottish Independence referendum, individuals intending to vote for separatism based their decision on perceptions that Scots had limited job prospects, were economically disadvantaged and had lower representation compared to their English counterparts. In 2014, the referendum resulted in 44.7% of the electorate (out of 84.6%) voting in favour of Scottish national independence, thereby sustaining the ongoing independence debate.

The strategy that is employed in a given time depends on how the group perceives the actions of the outgroup, in addition to efficacy and legitimacy (Haslam & Reicher, 2012). Therefore, in this study, we introduced and tested the role of perceived repression, alongside political trust, efficacy and legitimacy. We examined whether low political trust and strong perceived protest repression are associated with confrontational collective action through legitimacy and efficacy within the context of the Scottish Independence Movement.

### Method

We collected data from individuals who self‐identify as Scottish. Participants were recruited from Prolific, where members receive compensation for participating in online research. The data collection took place in March 2022. Prolific's pre‐screening criteria were utilized to ensure that only individuals aged 18 years and over, residing in Scotland and nationally identifying themselves as Scottish could participate in the study. After removing two participants who did not pass the attention check question, the final sample consisted of 304 participants. Among the participants, 150 self‐identified as male, 150 as female and 4 as other or preferred not to answer. The age range of the participants varied from 19 to 74 (*M* = 39.90, SD = 12.75). Regarding education, 55 participants had completed a postgraduate degree, 143 had a graduate degree, 103 had completed upper‐secondary or post‐secondary school, and one had completed primary school. A power analysis using Shiny app *pwrSEM* (Wang & Rhemtulla, 2021) indicated that the study achieved sufficient power (>.80) to detect effect sizes for all parameters in the models.

### Measures

We employed 5‐point Likert‐type multi‐item measures for all variables. All variables were operationalized as latent variables in SEM. For simplicity in interpretation and to explore correlations among variables, we also provide reliability coefficients for composite scores, created by averaging the items within each scale. The scales for *political trust* in British authorities and institutions (*α* = .91), *legitimacy of political violence* (*α* = .96), *efficacy of confrontational tactics* (*α* = .93) and *confrontational collective action intention* (*α* = .92) were consistently measured using the same scales as in Study 3.

#### Perceived protest repression

We measured perceived repression regarding protests with seven items adapted from Saavedra and Drury (2019). The sample item reads ‘*In the United Kingdom, some people are arrested without any more justification than having participated in a protest*’ (*α* = .94).

### Results and discussion

**FIGURE 3:**
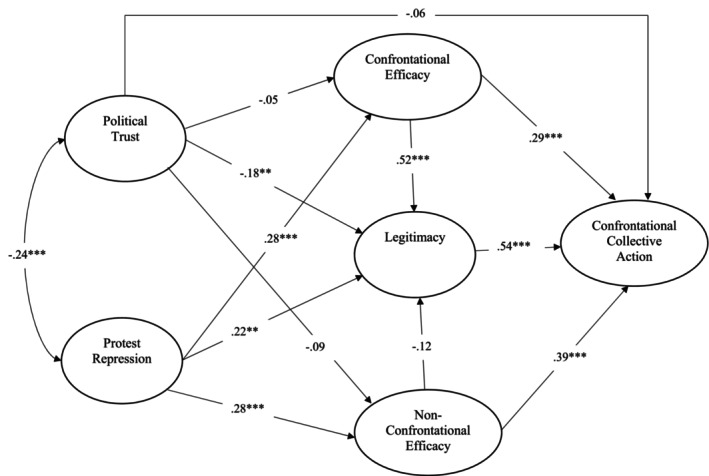
Summary of structural equation model testing, Study. **p < .01, ***p < .001.

The descriptive statistics and zero‐order correlations among our variables are provided in Table 4. Subsequently, we employed SEM analysis to assess and validate our model (see Figure 3), utilizing the *lavaan* package in *R* (Rosseel, 2012) for testing the proposed model.

**TABLE 4:** Means, standard deviations and correlations of all measures, Study 3.

	*M* (SD)	1	2	3	4	5	6
1. Political trust	2.01 (.80)	–	−.41***	−.27***	−.25***	−.13*	−.27***
2. Protest repression	2.78 (.97)		–	.35***	.27***	.28***	.44***
3. Legitimacy of protest violence	1.85 (.89)			–	.03	.53***	.64***
4. Efficacy of non‐confrontational tactics	3.67 (.92)				–	.06	.23***
5. Efficacy of confrontational tactics	1.64 (.86)					–	.50***
6. Confrontational collective action intention	1.81 (.91)						–

*Note*: *p < .05, ***p < .001.

The fit indices indicated that our model exhibited a good fit with the data: *χ* ^2^ = 971.850; df = 337; *p* < .001; CFI = .923; TLI = .913; RMSEA = .079, CI [.073, .085]; and *SRMR* = .064. As illustrated in Figure 3, a negative covariance existed between political trust and perceived protest repression (*b* = −.24, SE = .04, *p* < .001). Once again, political trust did not predict confrontational collective action (*b* = −.06, SE = .06, *p* = .364). Legitimacy of protest violence was predicted by lower political trust (*b* = −.18, SE = .06, *p* = .005), stronger protest repression (*b* = .22, SE = .06, *p* < .001) and greater efficacy of confrontational tactics (*b* = .52, SE = .07, *p* < .001). The efficacy of confrontational collective action was predicted by stronger protest repression (*b* = .25, SE = .06, *p* < .001). However, political trust did not predict the efficacy of confrontational tactics (*b* = −.05, SE = .06, *p* = .413). Lastly, greater willingness to participate in confrontational action was predicted by stronger legitimacy of protest violence (*b* = .54, SE = .06, *p* < .001) and greater efficacy of confrontational tactics (*b* = .29, SE = .07, *p* < .001). We also tested the role of the efficacy of non‐confrontational tactics on confrontational collective action. Confrontational collective action was predicted by the efficacy of non‐confrontational tactics too (*b* = .37, SE = .08, *p* < .001).

As in Study 1, we conducted an alternative model where we switched the roles of efficacy and legitimacy for a robustness check. In the alternative model, efficacy beliefs are predicted by legitimacy, as opposed to the original model where efficacy beliefs predicted legitimacy. Results showed that the original model had a lower *AIC* and *χ* ^*2*^ and higher *GFI*, suggesting that the alternative model showed a worse fit than the proposed model. Hence, Study 3 showed that tactic legitimacy is more likely to be predicted by tactic efficacy, compared to the other way around (see Data S1).

Subsequently, we conducted mediation analyses to examine the indirect pathway of perceived protest repression on willingness to participate in confrontational collective action through the efficacy of confrontational tactics and legitimacy of protest violence, using a bootstrapping method with 10,000 repetitions. Perceived protest repression predicted willingness to participate in confrontational collective action through tactic efficacy, *b* = .14, SE = .04, 95% CI [.08, .23], *p* < .001. Similarly, the legitimacy of protest violence mediated the relationship between perceived protest repression and confrontational collective action, *b* = .20, SE = .04, 95% CI [.15, .33], *p* < .001. Finally, we test whether low political trust predicts confrontational collective action through heightened legitimacy of protest violence. Although political trust did not predict confrontational collective action (*b* = −.11, SE = .06, *p* = .089), it indirectly predicts through heightened legitimacy of protest violence, *b* = −.23, SE = .05, 95% CI [−.18, .25], *p* < .001.

Overall, this study reveals that lower political trust was associated with greater perceived protest repression and higher legitimacy of protest violence. While lower political trust did not directly predict the efficacy of confrontational tactics, it is indirectly associated with a stronger willingness to participate in confrontational action through the heightened legitimacy of protest violence. Stronger efficacy of confrontational tactics was associated with greater willingness to engage in confrontational action. Furthermore, both tactic efficacy and legitimacy mediate the relationship between perceived protest repression and willingness to participate in confrontational collective action.

## GENERAL DISCUSSION

We explored the social‐psychological underpinnings of confrontational collective action across different political contexts by examining the roles of political trust, perceived protest repression, legitimacy of political and protest violence and efficacy of confrontational and non‐confrontational actions. Drawing from three studies conducted in Germany, Turkey and the United Kingdom, we found that the perceived efficacy and legitimacy of confrontational tactics consistently emerged as potent predictors of confrontational collective action across all contexts (see Table 5).

**TABLE 5:** Summary of results.

Hypotheses	Study 1 (Germany)	Study 2 (Kurds in Turkey)	Study 3 (Scots in the United Kingdom)
H1: Trust → Confrontational Collective Action (+)	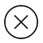	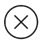	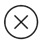
H2: Trust → Confrontational Efficacy (−)	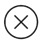		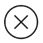
H3: Trust → Legitimacy of Violence (−)	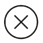		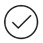
H4: Trust → Non‐Confrontational Efficacy (+)	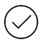		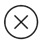
H5: Confrontational Efficacy → Legitimacy of Violence (+)	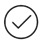		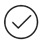
H6: Legitimacy of Violence → Confrontational Collective Action (+)	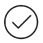	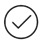	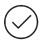
H7: Confrontational Efficacy → Confrontational Collective Action (+)	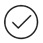	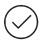	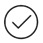
H8: Non‐Confrontational Efficacy → Legitimacy of Violence (−)	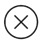		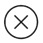
H9: Political Trust ↔ Protest Repression (−)			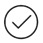
H10: Protest Repression → Confrontational Efficacy (+)			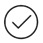
H11: Protest Repression → Legitimacy of Violence (+)			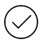

*Note*: Empty columns show that the respective hypothesis was not tested in the study.

The ‘nothing‐to‐lose’ hypothesis posits that individuals are more likely to engage in confrontational action when non‐confrontational (or moderate) action is perceived as a low‐efficacy strategy (Becker & Tausch, 2015; Jiménez‐Moya et al., 2015; Saab et al., 2016; Tausch et al., 2011). This perspective assumes that confrontational actions emerge as a last resort, driven by desperation or a perceived absence of viable alternatives. However, our findings challenge this notion in several ways. First, across all three studies, participants consistently rated the efficacy of non‐confrontational action as higher than that of confrontational action. This is inconsistent with the nothing‐to‐lose hypothesis, which would predict low non‐confrontational efficacy as a necessary precursor for confrontational action. Instead, our data show that confrontational collective action is predicted by the perceived efficacy of confrontational tactics themselves, both directly and indirectly via the legitimacy of protest violence. This indicates that confrontational actions are not merely reactive strategies adopted when non‐confrontational actions are deemed ineffective but can be deliberate, strategic choices rooted in their own perceived effectiveness and legitimacy in achieving collective action goals. Second, our findings on political trust further challenge the nothing‐to‐lose hypothesis. If confrontational actions were indeed a ‘last resort’ strategy employed when other political avenues are blocked or ineffective, as Tausch et al. (2011) argued that low efficacy indicates low political trust, we would expect a significant negative relationship between political trust and confrontational collective action. However, across all three studies, we found no evidence to support this relationship. This suggests that low political trust is not a necessary driver of confrontational collective action, and such actions are not simply the result of perceived political helplessness within democratic institutions. Third, our results suggest that the relatively higher efficacy of confrontational tactics can coexist with the relatively higher efficacy of non‐confrontational tactics (see Study 2), suggesting these strategies are not mutually exclusive. Rather than being framed as ‘last‐resort’ measures, confrontational tactics are part of a broader repertoire of collective action, selected based on their perceived suitability for achieving group objectives in specific contexts. This finding aligns with the idea of a continuum of action strategies, where groups dynamically calibrate their approaches depending on the interplay of efficacy, legitimacy and situational factors (Uysal et al., 2025; Uysal, Saavedra, & Drury, 2024).

The findings highlight the importance of understanding efficacy through specific actions that are committed to achieving ingroup‐determined goals. Traditional models of collective action in social psychology have largely focused on non‐confrontational actions, often overlooking the strategic use of confrontational tactics. This oversight is accompanied by the conceptualization of efficacy in a way that emphasizes group efficacy without clearly linking it to group goals and actions, or by focusing on the effectiveness of exclusively singular non‐confrontational actions while disregarding the multiple tactics employed by activists within the same movement. However, to clearly grasp the role of efficacy in collective action, it is necessary to focus on the efficacy of multiple tactics that are clearly conceptualized within group goals. Our results showed that the perceived efficacy of confrontational tactics to achieve group goals robustly predicts engagement in such actions, aligning with Hamann et al. (2024), which argues for the importance of linking efficacy beliefs to specific agents, actions and aims.

Moreover, the role of political trust in confrontational collective action has been complex and debated. Some studies suggest that a lack of options for non‐confrontational collective action motivates confrontational tactics (Jiménez‐Moya et al., 2015; Saab et al., 2016); therefore, confrontational tactics should be seen as more feasible where low political trust and low efficacy of non‐confrontational tactics are prevalent. Our findings offer a more nuanced perspective: while low political trust did not directly predict confrontational action, it contributes to perceptions of heightened protest repression and the legitimacy of confrontational tactics. Furthermore, perceived protest repression predicted willingness to participate in confrontational collective action through its legitimacy and efficacy.

Our findings demonstrate that the legitimacy of confrontational actions can be predicted by the perceived efficacy of these actions. This suggests that efficacy and legitimacy are deeply interconnected in the context of collective action. The perceived efficacy of an action in achieving its intended goals can enhance its legitimacy within the group. This aligns with the previous works that showed that actions seen as capable of realizing the group's objectives gain political credibility and acceptance (Hoerst & Drury, 2023; Jiménez‐Moya et al., 2019). In practical terms, when group members believe that confrontational tactics such as disruptive protests or civil disobedience are effective in achieving group goals, such as drawing attention to critical issues or pressuring authorities, these tactics are deemed more legitimate. This perceived legitimacy, alongside perceived efficacy, predicts the intention to engage in such actions. However, it is important to recognize that this interplay is context‐dependent, and there may be situations where high efficacy does not align with high legitimacy, or vice versa. It is necessary to remain open to the possibility that, in certain contexts, the levels of legitimacy and efficacy of actions may be incongruent. This highlights the need to examine these relationships across different contexts, using qualitative and person‐centred approaches to explore the nuances further.

Our research has several limitations that should be addressed in future studies. First, our studies were correlational in nature, which limits the ability to make causal inferences about the relationships between perceived efficacy, legitimacy and engagement in confrontational collective action. Nonetheless, our robustness check analyses with alternative model testing in Studies 1 and 3 showed the proposed models indicated a better fit than alternative models, suggesting the proposed set of relationships between legitimacy‐efficacy‐collective actions is more likely to be the case. Second, while our research highlights the importance of efficacy and legitimacy in predicting confrontational collective action, there is a need to probe into the sources of efficacy in social movements and collective action. Understanding how activists derive their sense of efficacy—from factors such as ingroup power, outgroup vulnerability, solidarity and social support—requires more naturalistic and qualitative approaches. Ethnographic and qualitative studies, in addition to participatory research practices that prioritize co‐production with activists themselves, can provide richer insights into the processes and experiences that emerge and endure efficacy in the context of sustained collective action. Third, future research should also explore how activists debate various tactics during social movements. It is crucial to understand how ingroup conflicts are managed and how consensual determination of group goals and legitimate and effective tactics are achieved. This calls, once again, for more realistic and natural research settings, such as longitudinal ethnographies that adopt participatory research practices (see Vestergren et al., 2018), which can capture the dynamic and evolving nature of sustained collective action.

## CONCLUSION

Our findings challenge the traditional notion that confrontational collective actions emerge solely as a last resort when non‐confrontational options are perceived as ineffective or unavailable. Across three diverse political contexts, we consistently found that the perceived efficacy of confrontational tactics and the legitimacy of protest violence were the strongest predictors of willingness to engage in confrontational collective action. In contrast, the low efficacy of non‐confrontational tactics and low political trust did not directly drive confrontational action, though low political trust predicted a heightened perception of protest repression and the legitimacy of protest violence. Therefore, it is argued that confrontational collective action did not arise solely from desperation, a lack of alternatives or political helplessness; rather, people engage in such actions when they are seen as a legitimate and effective tool for collective objectives and social change. Importantly, our results suggest that confrontational and non‐confrontational strategies are not mutually exclusive; individuals may view both as effective tools within a broader repertoire of collective action and employ them simultaneously depending on what the political and intergroup context requires.

## AUTHOR CONTRIBUTIONS

**Mete Sefa Uysal:** Conceptualization; methodology; data curation; formal analysis; project administration; visualization; funding acquisition; writing – original draft; writing – review and editing; resources. **John Drury:** Conceptualization; funding acquisition; writing – review and editing; supervision. **Yasemin Gülsüm Acar:** Conceptualization; writing – review and editing.

## FUNDING INFORMATION

Studies 2 and 3 were supported by the International Society of Political Psychology Scholars Under Threat Committee.

## CONFLICT OF INTEREST STATEMENT

The authors declare no conflict of interest.

## Supporting information


Data S1.

## Data Availability

The datasets and analysis codes are openly accessible at https://osf.io/zysf7/?view_only=59107f659aa2422eb3c04a337f1ff6e8.

## References

[bjso12891-bib-0001] Abrams, D. , & Hogg, M. A. (1987). Language attitudes, frames of reference, and social identity: A Scottish dimension. Journal of Language and Social Psychology, 6(3–4), 201–213. 10.1177/0261927X8763004

[bjso12891-bib-0002] Abrams, D. , Travaglino, G. A. , Grant, P. R. , Templeton, A. , Bennett, M. , & Lalot, F. (2020). Mobilizing IDEAS in the Scottish referendum: Predicting voting intention and well‐being with the identity‐deprivation‐efficacy‐action‐subjective well‐being model. British Journal of Social Psychology, 59(2), 425–446. 10.1111/bjso.12355 31746019 PMC7186818

[bjso12891-bib-0004] Acar, Y. G. , & Uluğ, Ö. M. (2022). When and why does political trust predict well‐being in authoritarian contexts? Examining the role of political efficacy and collective action among opposition voters. British Journal of Social Psychology, 61(3), 861–881. 10.1111/bjso.12505 34724227

[bjso12891-bib-0003] Acar, Y. G. , Sandal‐Önal, E. , Şen, E. , & Uysal, M. S. (2025). Studying Kurdishness in Turkey: A review of existing research. British Journal of Social Psychology, 64(1), e12842. 10.1111/bjso.12842 39817618

[bjso12891-bib-0005] Adam‐Troian, J. , Çelebi, E. , & Mahfud, Y. (2020). “Return of the repressed”: Exposure to police violence increases protest and self‐sacrifice intentions for the yellow vests. Group Processes & Intergroup Relations, 23(8), 1171–1186. 10.1177/1368430220920707

[bjso12891-bib-0006] Agostini, M. , & van Zomeren, M. (2021). Toward a comprehensive and potentially cross‐cultural model of why people engage in collective action: A quantitative research synthesis of four motivations and structural constraints. Psychological Bulletin, 147(7), 667–700. 10.1037/bul0000256 34855427

[bjso12891-bib-0008] Ayanian, A. H. , & Tausch, N. (2016). How risk perception shapes collective action intentions in repressive contexts: A study of Egyptian activists during the 2013 post‐coup uprising. British Journal of Social Psychology, 55(4), 700–721. 10.1111/bjso.12164 27696433

[bjso12891-bib-0007] Ayanian, A. H. , Adra, A. , Zeineddine, F. B. , Saab, R. , Vollhardt, J. R. , & Tausch, N. (2025). Collective action under repressive conditions: Integration of individual, group, and structural level research, recommendations, and reflections. Social Issues and Policy Review, 19(1), e70000. 10.1111/sipr.70000

[bjso12891-bib-0009] Ayanian, A. H. , Tausch, N. , Acar, Y. G. , Chayinska, M. , Cheung, W.‐Y. , & Lukyanova, Y. (2021). Resistance in repressive contexts: A comprehensive test of psychological predictors. Journal of Personality and Social Psychology, 120(4), 912–939. 10.1037/pspi0000285 32614221

[bjso12891-bib-0010] Becker, J. C. , & Tausch, N. (2015). A dynamic model of engagement in normative and non‐normative collective action: Psychological antecedents, consequences, and barriers. European Review of Social Psychology, 26(1), 43–92. 10.1080/10463283.2015.1094265

[bjso12891-bib-0011] Boykoff, J. (2006). The suppression of dissent: How the state and mass media squelch US American social movements. Routledge.

[bjso12891-bib-0070] Cagaptay, S. (2004). Race, assimilation and Kemalism: Turkish nationalism and the minorities in the 1930s. Middle Eastern Studies, 40(3), 86–101. 10.1080/0026320042000213474

[bjso12891-bib-0012] Devine, D. , Gaskell, J. , Jennings, W. , & Stoker, G. (2020). Exploring trust, mistrust and distrust: Discussion paper for the 1st Digital Workshop of the ESRC ‘TrustGov’ project. In *Working Paper Series*. https://drive.google.com/file/d/1C5joqTyly7XGnHsWt3SnwCM7q2WH3Ful/view

[bjso12891-bib-0013] Drury, J. , & Reicher, S. (1999). The intergroup dynamics of collective empowerment: Substantiating the social identity model of crowd behavior. Group Processes & Intergroup Relations, 2(4), 381–402. 10.1177/1368430299024005

[bjso12891-bib-0014] Drury, J. , & Reicher, S. (2000). Collective action and psychological change: The emergence of new social identities. British Journal of Social Psychology, 39(4), 579–604. 10.1348/014466600164642 11190686

[bjso12891-bib-0069] Drury, J. , & Reicher, S. (2009). Collective psychological empowerment as a model of social change: Researching crowds and power. Journal of Social Issues, 65(4), 707–725. 10.1111/j.1540-4560.2009.01622.x

[bjso12891-bib-0015] Drury, J. , Reicher, S. D. , & Stott, C. (2012). The psychology of collective action: Crowds and change. In B. Wagoner , E. Jensen , & J. A. Oldmeadow (Eds.), Culture and social change: Transforming society through the power of ideas (pp. 19–38). IAP Information Age Publishing.

[bjso12891-bib-0016] Francisco, R. A. (1995). The relationship between coercion and protest: An empirical evaluation in three coercive states. Journal of Conflict Resolution, 39(2), 263–282. 10.1177/0022002795039002003

[bjso12891-bib-0017] GESIS—Leibniz‐Institut für Sozialwissenschaften . (2019). ALLBUS/GGSS 2018 (Allgemeine Bevölkerungsumfrage der Sozialwissenschaften/German General Social Survey 2018) (Cologne. ZA5270 Data file Version, 2.0.0). GESIS Data Archive.

[bjso12891-bib-0018] Grant, P. R. , Bennett, M. , & Abrams, D. (2017). Using the SIRDE model of social change to examine the vote of Scottish teenagers in the 2014 independence referendum. British Journal of Social Psychology, 56(3), 455–474. 10.1111/bjso.12186 28144960

[bjso12891-bib-0019] Greijdanus, H. , De Matos Fernandes, C. A. , Turner‐Zwinkels, F. , Honari, A. , Roos, C. A. , Rosenbusch, H. , & Postmes, T. (2020). The psychology of online activism and social movements: Relations between online and offline collective action. Current Opinion in Psychology, 35, 49–54. 10.1016/j.copsyc.2020.03.003 32330859

[bjso12891-bib-0020] Grigoriadis, I. N. (2016). The peoples' democratic party (HDP) and the 2015 elections. Turkish Studies, 17(1), 39–46. 10.1080/14683849.2015.1136086

[bjso12891-bib-0021] Gulliver, R. , Chan, C. S. , Tam, Y. , Lau, I. S. , Hong, Y. Y. , & Louis, W. R. (2023). Political distrust, perceived threat, and intentions to engage in normative and violent collective action: A mixed‐methods study. European Journal of Social Psychology, 53(2), 401–417. 10.1002/ejsp.2910

[bjso12891-bib-0022] Güneş, C. (2019). The Kurds in a new Middle East: The changing of a regional conflict. Palgrave Macmillan.

[bjso12891-bib-0023] Hamann, K. R. S. , Wullenkord, M. C. , & Reese, G. (2024). Believing that we can change our world for the better: A triple‐a (agent‐action‐aim) framework of self‐efficacy beliefs in the context of collective social and ecological aims. Personality and Social Psychology Review, 28(1), 11–53. 10.1177/10888683231178056 37386819 PMC10851658

[bjso12891-bib-0024] Haslam, S. A. , & Reicher, S. D. (2012). When prisoners take over the prison: A social psychology of resistance. Personality and Social Psychology Review, 16(2), 154–179. 10.1177/1088868311419864 21885855

[bjso12891-bib-0025] Hetherington, M. J. (2005). Why trust matters: Declining political trust and the demise of American liberalism. Princeton University Press.

[bjso12891-bib-0026] Hetherington, M. J. , & Husser, J. A. (2012). How trust matters: The changing political relevance of political trust. American Journal of Political Science, 56(2), 312–325. 10.1111/j.1540-5907.2011.00548.x

[bjso12891-bib-0027] Hoerst, C. , & Drury, J. (2023). Disrupting a (far‐right) mobilisation discourages bystander support by decreasing perceived organisational efficacy and legitimacy.

[bjso12891-bib-0028] Honari, A. (2018). From ‘the effect of repression’ toward ‘the response to repression’. Current Sociology, 66(6), 950–973. 10.1177/0011392118787585 30270933 PMC6146312

[bjso12891-bib-0029] Jackson, J. , Huq, A. Z. , Bradford, B. , & Tyler, T. R. (2013). Monopolizing force? Police legitimacy and public attitudes toward the acceptability of violence. Psychology, Public Policy, and Law, 19(4), 479–497. 10.1037/a0033852

[bjso12891-bib-0030] Jiménez‐Moya, G. , Miranda, D. , Drury, J. , Saavedra, P. , & González, R. (2019). When nonactivists care: Group efficacy mediates the effect of social identification and perceived instability on the legitimacy of collective action. Group Processes & Intergroup Relations, 22(4), 563–577. 10.1177/1368430217751631

[bjso12891-bib-0031] Jiménez‐Moya, G. , Spears, R. , Rodríguez‐Bailón, R. , & de Lemus, S. (2015). By any means necessary? When and why low group identification paradoxically predicts radical collective action. Journal of Social Issues, 71(3), 517–535. 10.1111/josi.12126

[bjso12891-bib-0032] Koc, I. , Hancioglu, A. , & Cavlin, A. (2008). Demographic differentials and demographic integration of Turkish and Kurdish populations in Turkey. Population Research and Policy Review, 27(4), 447–457. 10.1007/s11113-008-9072-y

[bjso12891-bib-0033] Kogler, C. , Batrancea, L. , Nichita, A. , Pantya, J. , Belianin, A. , & Kirchler, E. (2013). Trust and power as determinants of tax compliance: Testing the assumptions of the slippery slope framework in Austria, Hungary, Romania and Russia. Journal of Economic Psychology, 34, 169–180. 10.1016/j.joep.2012.09.010

[bjso12891-bib-0034] Levi, M. , & Stoker, L. (2000). Political trust and trustworthiness. Annual Review of Political Science, 3, 475–507. 10.1146/annurev.polisci.3.1.475

[bjso12891-bib-0035] Li, J.‐B. , & Finkenauer, C. (2021). Hong Kong university students' normative beliefs about aggression toward police during social protests 2019–2020: The role of ecological risks and future orientation. Crime & Delinquency, 69(11), 2274–2302. 10.1177/00111287211014145

[bjso12891-bib-0036] Li, L. (2011). Distrust in government leaders, demand for leadership change, and preference for popular elections in rural China. Political Behavior, 33(2), 291–311. 10.1007/s11109-010-9111-3

[bjso12891-bib-0037] Lichbach, M. I. (1987). Deterrence or escalation? The puzzle of aggregate studies of repression and dissent. Journal of Conflict Resolution, 31(2), 266–297. 10.1177/0022002787031002003

[bjso12891-bib-0038] Marazzi, C. , & Vollhardt, J. R. (2025). “When you live in a colony… every act counts”: Exploring engagement in and perceptions of diverse anti‐colonial resistance strategies in Puerto Rico. British Journal of Social Psychology, 64(2), e12808. 10.1111/bjso.12808 39382120 PMC11927381

[bjso12891-bib-0039] McAdam, D. (1990). Freedom summer. Oxford University Press.

[bjso12891-bib-0040] Moghadam, V. , & Gheytanchi, E. (2011). Political opportunities and strategic choices: Comparing feminist campaigns in Morocco and Iran. Mobilization, 15(3), 267–288. 10.17813/maiq.15.3.n248564371645v14

[bjso12891-bib-0041] Mummendey, A. , Kessler, T. , Klink, A. , & Mielke, R. (1999). Strategies to cope with negative social identity: Predictions by social identity theory and relative deprivation theory. Journal of Personality and Social Psychology, 76(2), 229–245. 10.1037/0022-3514.76.2.229 10074707

[bjso12891-bib-0042] Mutlu, S. (1996). Ethnic Kurds in Turkey: A demographic study. International Journal of Middle East Studies, 28(4), 517–541. 10.1017/S0020743800063819

[bjso12891-bib-0043] Penić, S. , Donnay, K. , Bhavnani, R. , Elcheroth, G. , & Albzour, M. (2024). How does the geography of surveillance affect collective action? Political Psychology, 45(2), 319–340. 10.1111/pops.12925

[bjso12891-bib-0044] Prentice, D. , & Paluck, E. L. (2020). Engineering social change using social norms: Lessons from the study of collective action. Current Opinion in Psychology, 35, 138–142. 10.1016/j.copsyc.2020.06.012 32746001

[bjso12891-bib-0046] Reicher, S. D. (1996). “The Battle of Westminster”: Developing the social identity model of crowd behaviour in order to explain the initiation and development of collective conflict. European Journal of Social Psychology, 26(1), 115–134. 10.1002/(SICI)1099-0992(199601)26:1<115::AID-EJSP740>3.0.CO;2-Z

[bjso12891-bib-0045] Reicher, S. , & Haslam, S. A. (2006). Rethinking the psychology of tyranny: The BBC prison study. British Journal of Social Psychology, 45(1), 1–40. 10.1348/014466605X48998 16573869

[bjso12891-bib-0047] Rosseel, Y. (2012). Lavaan: An R package for structural equation modeling. Journal of Statistical Software, 48(2), 1–36. 10.18637/jss.v048.i02

[bjso12891-bib-0048] Saab, R. , Spears, R. , Tausch, N. , & Sasse, J. (2016). Predicting aggressive collective action based on the efficacy of peaceful and aggressive actions. European Journal of Social Psychology, 46(5), 529–543. 10.1002/ejsp.2193

[bjso12891-bib-0049] Saavedra, P. , & Drury, J. (2019). Including political context in the psychological analysis of collective action: Development and validation of a measurement scale for subjective political openness. Journal of Social and Political Psychology, 7(2), 665–694. 10.5964/jspp.v7i2.1030

[bjso12891-bib-0050] Scheepers, D. , Spears, R. , Doosje, B. , & Manstead, A. S. R. (2006). Diversity in in‐group bias: Structural factors, situational features, and social functions. Journal of Personality and Social Psychology, 90(6), 944–960. 10.1037/0022-3514.90.6.944 16784344

[bjso12891-bib-0051] Şen, E. , Sandal‐Önal, E. , Uysal, M. S. , & Acar, Y. G. (2023). The political psychology of Kurds in Turkey: Critical perspectives on identity, narratives, and resistance. Palgrave Macmillan.

[bjso12891-bib-0052] Stott, C. , & Reicher, S. (1998). Crowd action as intergroup process: Introducing the police perspective. European Journal of Social Psychology, 28(4), 509–529. 10.1002/(SICI)1099-0992(199807/08)28:4<509::AID-EJSP877>3.0.CO;2-C

[bjso12891-bib-0053] Tajfel, H. , & Turner, J. C. (1979). An integrative theory of intergroup conflict. In W. G. Austin & S. Worchel (Eds.), The social psychology of intergroup relations (pp. 33–37). Brooks/Cole.

[bjso12891-bib-0054] Tausch, N. , Becker, J. C. , Spears, R. , Christ, O. , Saab, R. , Singh, P. , & Siddiqui, R. N. (2011). Explaining radical group behavior: Developing emotion and efficacy routes to normative and nonnormative collective action. Journal of Personality and Social Psychology, 101(1), 129–148. 10.1037/a0022728 21500925

[bjso12891-bib-0068] Thomas, E. F. , Mavor, K. I. , & McGarty, C. (2012). Social identities facilitate and encapsulate action‐relevant constructs. Group Processes & Intergroup Relations, 15(1), 75–88. 10.1177/1368430211413619

[bjso12891-bib-0055] Uysal, M. S. , Acar, Y. G. , Sabucedo, J. M. , & Cakal, H. (2022). To participate or not participate, that's the question: The role of moral obligation and different risk perceptions on collective action. Journal of Social and Political Psychology, 10(2), 445–459. 10.5964/jspp.7207

[bjso12891-bib-0056] Uysal, M. S. , Martinez, N. , & Vestergren, S. (2025). The horror of today and the terror of tomorrow: The role of future existential risks and present‐day political risks in climate activism. British Journal of Social Psychology, 64(1), e12821. 10.1111/bjso.12821 39494606 PMC11590411

[bjso12891-bib-0057] Uysal, M. S. , Saavedra, P. , & Drury, J. (2024). Beyond normative and non‐normative: A systematic review on predictors of confrontational collective action. British Journal of Social Psychology, 63(3), 1385–1409. 10.1111/bjso.12735 38390962

[bjso12891-bib-0058] Uysal, M. S. , Şen, E. , Sandal‐Önal, E. , & Acar, Y. G. (2024a). Addressing epistemic violence and methodological nationalism through a meta‐analytical review on intergroup contact and conflict studies in Turkey. Journal of Social and Political Psychology, 12(2), 225–246. 10.5964/jspp.13241

[bjso12891-bib-0059] Uysal, M. S. , Şen, E. , Sandal‐Önal, E. , & Acar, Y. G. (2024b). Researching identity in the imposed boundaries of a nation‐state: A meta‐analytical review of identity and intergroup relations between Kurds and Turks. Peace and Conflict: Journal of Peace Psychology, 20, 764. 10.1037/pac0000764

[bjso12891-bib-0060] Uysal, M. S. , Vestergren, S. , Varela, M. , & Lindner, C. (2024). “System change not climate change”: Effective environmental policies and state repression moderate the relationship between psychological predictors and environmental collective action. Global Environmental Psychology, 2, e11259. 10.5964/gep.11259

[bjso12891-bib-0061] van Zomeren, M. , Postmes, T. , & Spears, R. (2008). Toward an integrative social identity model of collective action: A quantitative research synthesis of three socio‐psychological perspectives. Psychological Bulletin, 134(4), 504–535. 10.1037/0033-2909.134.4.504 18605818

[bjso12891-bib-0062] van Zomeren, M. , Saguy, T. , & Schellhaas, F. M. H. (2013). Believing in “making a difference” to collective efforts: Participative efficacy beliefs as a unique predictor of collective action. Group Processes & Intergroup Relations, 16(5), 618–634. 10.1177/1368430212467476

[bjso12891-bib-0063] van Zomeren, M. , Spears, R. , & Leach, C. W. (2010). Experimental evidence for a dual pathway model analysis of coping with the climate crisis. Journal of Environmental Psychology, 30(4), 339–346. 10.1016/j.jenvp.2010.02.006

[bjso12891-bib-0064] Vestergren, S. , Drury, J. , & Chiriac, E. H. (2018). How collective action produces psychological change and how that change endures over time: A case study of an environmental campaign. British Journal of Social Psychology, 57(4), 855–877. 10.1111/bjso.12270 30079590 PMC6220852

[bjso12891-bib-0065] Wang, Y. A. , & Rhemtulla, M. (2021). Power analysis for parameter estimation in structural equation modeling: A discussion and tutorial. Advances in Methods and Practices in Psychological Science, 4(1), 2515245920918253. 10.1177/2515245920918253

[bjso12891-bib-0066] Wood, L. J. (2007). Breaking the wave: Repression, identity, and Seattle tactics. Mobilization, 12(4), 377–388. 10.17813/maiq.12.4.a38x78203j3502q0

[bjso12891-bib-0067] Zúñiga, C. , Asún, R. , & Louis, W. (2023). Normative and non‐normative collective action facing repression in a democratic context: A mixed study in a Chilean social movement. Journal of Social and Political Psychology, 11(1), 362–382. 10.5964/jspp.7973

