# Supplementary material for: ‘Nothing to lose or a world to win’: Reconsidering efficacy, legitimacy, political trust and repression in confrontational collective action
Source: Br J Soc Psychol. 2025 Apr 28;64(3):e12891. doi: 10.1111/bjso.12891 (PMC12038084; doi:10.1111/bjso.12891)
Supplement: Supplementary file 1 — Data S1. [file BJSO-64-0-s001.docx]

**Study 1: Control Variables**

*Social class* was measured with an item reads as “*What class would you describe yourself as belonging to?*” (1 = *lower*, 2 = *working*, 3 = *middle*, 4 = *upper-middle*, 5 = *upper*). Similarly, the *economic situation* was measured with an item that reads “*How would you rate your current financial situation*” (1 = *very bad*, 5 = *very good*). Last, *political orientation* was assessed by using a left-right spectrum self-placement item (1 = *left*, 10 = *right*).

**Study 1: Model comparison between original and alternative model**

Our data is correlational, and claims of directions between variables are speculative. Therefore, as a robustness check, we conducted an alternative model where we switched the roles of efficacy and legitimacy. In other words, in the alternative model, efficacy beliefs are predicted by legitimacy, as opposed to the original model where efficacy beliefs predicted legitimacy. Results showed that the alternative model fit the data worse than our proposed model (Original model: χ2 = 202.11, df = 9, p < .001, CFI = .964; GFI = .984; AGFI = .949; AIC = 41,923.991 vs. Alternative model: χ2 = 203.52, df = 9, p < .001, CFI = .963; GFI = .983; AGFI = .948; AIC = 41,925.497). As lower χ2, lower AIC, higher CFI, and higher GFI and AGFI indicate better fit, this suggests that tactic legitimacy is more likely to be predicted by tactic efficacy, rather than the other way around.

**Study 3: Model comparison between original and alternative model**

As in Study 1, we conducted an alternative model where we switched the roles of efficacy and legitimacy for a robustness check. In the alternative model, efficacy beliefs are predicted by legitimacy, as opposed to the original model where efficacy beliefs predicted legitimacy. Results showed that the alternative model fit the data worse than our proposed model (Original model: χ2 = 972.675, CFI = .923; GFI = .815; AGFI = .777; AIC = 16,712.910 vs. Alternative model: χ2 = 973.982, CFI = .923; GFI = .814; AGFI = .777; AIC = 16,714.218). Hence, Study 3 showed that tactic legitimacy is more likely to be predicted by tactic efficacy, compared to the other way around.
